# Social Inequalities in Breakfast Consumption among Adolescents in Spain: The DESKcohort Project

**DOI:** 10.3390/nu13082500

**Published:** 2021-07-22

**Authors:** Laura Esquius, Alicia Aguilar-Martínez, Marina Bosque-Prous, Helena González-Casals, Anna Bach-Faig, Ester Colillas-Malet, Gemma Salvador, Albert Espelt

**Affiliations:** 1 Foodlab Research Group, Faculty of Health Sciences, Universitat Oberta de Catalunya, 08018 Barcelona, Spain; lesquius@uoc.edu (L.E.); aaguilarmart@uoc.edu (A.A.-M.); abachf@uoc.edu (A.B.-F.); 2 Faculty of Health Sciences, Universitat Oberta de Catalunya, 08018 Barcelona, Spain; 3 Departament de Psicobiologia i Metodologia en Ciències de la Salut, Universitat Autònoma de Barcelona (UAB), 08193 Bellaterra, Spain; aespelt@umanresa.cat; 4 Department of Epidemiology and Public Health, Faculty of Health Sciences of Manresa, Universitat de Vic—Universitat Central de Catalunya (UVic-UCC), 08242 Manresa, Spain; HGonzalez@umanresa.cat (H.G.-C.); ecolillas@umanresa.cat (E.C.-M.); 5 Agència de Salut Pública de Catalunya, Departament de Salut, Generalitat de Catalunya, 08005 Barcelona, Spain; gemma.salvador@gencat.cat; 6 Centro de Investigación Biomédica en Red de Epidemiología y Salud Pública (CIBERESP), 28029 Madrid, Spain

**Keywords:** breakfast skipping, adolescents, socioeconomic position, social inequalities, social determinants of health

## Abstract

Breakfast has a critical role in energy balance and dietary regulation. Consequently, it is considered an important component of a healthy diet, especially in adolescence, when there are great opportunities to consolidate habits and establish future patterns of healthiness in adulthood. Socioeconomic position (SEP) causes inequalities that are reflected in health behaviors, physical activity, mental health, and diet. Therefore, we conducted a cross-sectional study using data from the 2019–2020 DESKcohort project (Spain) to explore the relationships between breakfast and sociodemographic characteristics, health-related behaviors, and school performance of 7319 adolescents. Our findings showed that the prevalence of skipping breakfast every day was 19.4% in girls and 13.7% in boys and was related to students’ SEP. The risk of skipping breakfast was 30% higher in girls from the most disadvantaged SEP, in comparison to those in the most advanced SEP (prevalence ratio (PR) = 1.30; 95% confidence interval (CI) = 1.11–1.54). Also, boys from the most disadvantaged SEP showed 28% higher risk of skipping breakfast than those in the most advanced SEP (PR = 1.28; 95% CI = 1.04–1.59). In conclusion, future public policies should be adapted considering a SEP and gender perspective to avoid increasing nutritional and health inequalities.

## 1. Introduction

Adolescence is an important period in life in which the opportunities for consolidating healthy lifestyles are great and future patterns of adult health are established [1]. During adolescence, individuals have increasing control over their food choices and dietary habits. In this context, families, peers, and schools play a relevant role and should be considered among the determinants of health [2,3]. Adolescence is, therefore, the right vital stage to develop health promotion programs aimed at influencing optimal growth and development, helping to reduce the chronic diseases in adulthood and indirectly favoring adequate academic performance [4,5,6,7,8].

Socioeconomic position (SEP) refers to the social and economic factors that influence what positions individuals or groups hold within the structure of a society. Such factors include educational level, income, and wealth. Different opportunities between people with a different SEP cause socioeconomic inequalities that are reflected in health [9]. In Spanish adolescents, the effects of social inequalities have been found in several diseases and health behaviors, including obesity, dental health, physical activity, mental health, and diet [10].

Breakfast, defined as the first meal of the day, has a critical role in energy balance and dietary regulation. Consequently, it is considered an important component of a healthy diet [11]. The metabolic effects of breakfast are an open question and the timing of breakfast is a relevant aspect since it is directly connected to nighttime fasting duration, which has been reported to be crucial for metabolism [12]. Currently, there is no consensus on the definition of breakfast. Such definitions vary across studies [11,13,14,15], either considering the time of consumption, the energy content, or the included foods and beverages. Moreover, for adolescent students in Mediterranean context, it is possible to have two eating occasions for breakfast, at home or at school. Different studies have focused on the analysis of breakfast and its quality considering any of the two eating occasions [16].

Children and adolescents who regularly consume breakfast more likely have a good diet quality [13,14,15,16,17]. Moreover, having breakfast has also been associated with positive effects on students’ cognitive development and better school performance in children [18,19,20,21]. However, breakfast is the meal that is most often skipped by children and adolescents [22]. As previously reviewed, a 10% to 30% prevalence of skipping breakfast was identified among adolescents which increased over age [23]. Skipping breakfast has been associated with female gender, later adolescence, living in single parent families, and lower socioeconomic position. Increased breakfast skipping has also been found to be positively correlated with unhealthy lifestyle, poorer diet, less physical activity, and more time spent watching television [17].

For children and adolescents, skipping meals (in particular breakfast, that stops nighttime fasting) could be a predictor of lifestyle behaviors, and it has been related to overweight, obesity, metabolic diseases [23] and it is a common eating behavior among adolescents that puts them at risk of nutrient deficiencies since it has been reported to decrease daily energy and nutrient intake [24]. As the HBSC study pointed out, there is an association between SEP and breakfast behaviors in adolescence, therefore daily breakfast consumptions should be encouraged as much as possible within the context of each country and family [17].

The main objective of this study was to estimate the prevalence of secondary-school students from Central Catalonia that regularly skip breakfast and analyze how social inequalities are related to this eating behavior.

## 2. Materials and Methods

This study uses a cross-sectional design with data from the first wave of the DESKcohort project [25], which was conducted between October 2019 and February 2020. The aim of the DESKcohort project was to monitor 12- to 18-year-old students that attend educational centers in Central Catalonia, collecting and analyzing data about health-related factors, and social and educational life. The 91 secondary education centers of Central Catalonia were invited to participate in the project, and 65 accepted (71%). The final study population consisted of 7319 students aged 12–18 years. During data collection, each participant responded to an online self-administered questionnaire using a tablet. This questionnaire addressed demographic, educational, and socioeconomic factors, and also health and health-related behaviors. The administration of the questionnaires by a trained person in the schools allowed clarifying concepts with the students both before and during the process.

The dependent variable was “skipping breakfast every day” and was constructed on the basis of the question “How many days have you had breakfast in the last week?” [26]. It had four possible response options: none; 1–3 days; 4–6 days; every day. Skipping breakfast every day was built as a dichotomous variable (never having breakfast vs. having breakfast some days or every day). We considered breakfast as the first meal of the day that breaks the fasting status after the long period of sleep. It occurs within 2 to 3 h of awakening, before starting classes. Finally, it comprises food or beverage from at least one food group, and may be consumed at any location [11]. Thus, according to this definition, skipping breakfast was considered as not eating any solid or liquid food on any day of the last week [24], and thus there is a skipping of all the breakfasts. 

The main independent variable was the perceived SEP, which was a continuous variable adapted from the validated MacArthur Scale of Subjective Social Status [27], which has excellent test-retest reliability (intraclass correlation = 0.73). To define this variable, we used the following question: “Think of this bar as representing where people stand in society. At the top (100) are the people who are the best off—those who have the most money, the most education and the most respected jobs. At the bottom (0) are the people who are the worst off—who have the least money, least education and the least respected jobs or no job. Where would you place your family on this scale?” The values ranged from 0 to 100, with higher values indicating a more advantaged socioeconomic position. The responses were finally divided in tertiles.

As independent variables, we also considered the following social factors: gender (girl or boy); age (years); course (second and fourth courses of compulsory secondary education (ISCED 2), and second course of post-compulsory secondary education and Intermediate Level Training Cycles (ISCED 3), according to the UNESCO International Standard Classification of Education) [28]; migratory status (native, first- or second-generation immigrant); size of municipality (≤5000 inhabitants, 5001–20,000 inhabitants, >20,000 inhabitants); and academic performance (good, average, or poor grades). Moreover, we adjusted the analyses for the following health-related variables: self-perceived health (responses were divided in the following categories: “excellent or very good” or “good, fair, or poor”, as few participants reported having a fair or poor health status); emotional state (continuous variable divided in tertiles); physical activity (reaching the WHO recommendations for physical activity in adolescents (>60 min per day), or not) [29]; body mass index (BMI) (underweight, normal weight, or overweight/obesity, defined using age- and sex-specific BMI cut-offs) [30]; and being on a diet (“no”, “yes, to lose or maintain weight”, and “yes, for other reasons”).

### Data Analysis

All analyses were conducted separately by gender. First, we described the main characteristics of the participants. Prevalence of skipping breakfast was estimated by each independent variable, with 95% confidence intervals (95% CI). To explore whether there were social inequalities in skipping breakfast among adolescents from Central Catalonia, we used multilevel Poisson regression models with robust variance, which yielded prevalence ratios (PR) and their corresponding 95% CI [31,32]. In the first multilevel model, we include only the variable “skipping breakfast every day”, to calculate the variability of the prevalence between educational centers. Then, we calculated the associations between skipping breakfast, the perceived SEP, and the other social variables. In each analysis, we included the dependent variable and only one independent variable in the multilevel model (crude models). Afterwards, we estimated the adjusted PR, including at the same time in the regression model the perceived SEP and the other social variables, and adjusting by the health-related variables. The final adjusted Poisson regression models for girls and boys included only the variables that were statistically significant (*p*-value < 0.05) in the multilevel multivariate analysis. All statistical analyses were conducted with STATA 16.

## 3. Results

Table 1 shows the characteristics of the study population divided by gender. Overall, around 52% of participants were girls. We observed no difference in age between girls and boys (mean = 15.3 years and SD = 1.7 for both). As for SEP, on a scale from 0 to 100, where higher values indicated more advantaged SEPs, the mean perceived SEP was not significantly different between girls and boys: 63.1 and 63.3, respectively (*p*-value = 0.398). With respect to the other social variables, we obtained the following results: 21% of girls and 19.4% of boys were migrants of first or second generations; around 40% of the participants lived in a municipality of 5001 to 20,000 inhabitants; and more than a half of the participants reported average grades (57.9% of the girls and 58.1% of the boys). Moreover, we collected the following health-related data: 57.4% of the people reported excellent or very good general health status (50.1% in girls and 65.3% in boys); 48.0% did not meet WHO (World Health Organization) recommendations on physical activity (57.7% in girls and 37.4% in boys); 18.2% were overweight or presented obesity (14.9% in girls and 21.7% in boys); and 11.7% were on a diet (12.7% in girls and 10.6% in boys). 

The prevalence of not having breakfast in the week before the questionnaire among adolescents was significantly higher in girls (19.4%; 95% CI = 18.2–20.7) than in boys (13.8%; 95% CI = 12.7–14.9) (*p*-value < 0.001). Moreover, the prevalence was significantly higher in adolescents from the lower tertile of SEP, in comparison to adolescents from the upper tertile: 24.5% of girls (95% CI = 22.3–26.9%) and 16.1% of boys (95% CI = 14.2–18.3%) from the lowest SEP tertile had skipped breakfast in the week before the questionnaire, versus 14.8% of girls (95% CI = 12.9–17.0) and 10.5% of boys (95% CI = 8.8–12.5%) from the highest SEP tertile (Table 2). In relation to the other social variables, a higher prevalence of skipping breakfast was observed in older students and in those with lower grades in both boys and girls. Likewise, in girls, a higher prevalence of skipping breakfast was observed in first- or second-generation migrants, in comparison to natives. However, no significant differences were observed between natives and migrants in boys. Finally, there were no differences in the prevalence of skipping breakfast on the basis of the size of the municipality, either among girls or boys. As for the health-related variables, the prevalence of skipping breakfast in both boys and girls was higher in the following subpopulations: adolescents reporting good, fair, or poor health; those in the lowest tertile of emotional state (i.e., with worse mood); the ones who did not reach WHO Physical Activity recommendations; and adolescents presenting overweight or obesity. No significant differences were found in relation to the state of being on a diet, both for girls and boys.

The results of the multilevel Poisson regression models with robust variance are presented in Table 3. In girls, the variance of skipping breakfast between educational centres was less than 1.8% and in boys less than 2.3%. In the model adjusted by different social and health-related variables (adjusted model in Table 3), the risk of skipping breakfast every day was 30% higher in girls from the most disadvantaged SEP, in comparison to those from the most advantaged SEP (PR = 1.30; 95% CI = 1.12–1.52). Similarly, it was 28% higher in boys from the most disadvantaged SEP, in comparison to the ones from the most advantaged SEP (PR = 1.28; 95% CI = 1.04–1.58). We also observed a statistically significant association between the course and the habit of skipping breakfast among girls: PR = 1.21 (95% CI = 1.00–1.45) in the fourth course of compulsory secondary education (CSE) (ISCED2); PR = 1.42 (95% CI = 1.19–1.71) in the second course of post-CSE (ISCED3); and PR = 1.52 (95% CI = 1.23–1.88) in the Intermediate Level Training Cycles (ISCED3), in comparison to the second course of CSE (ISCED2). A similar significant association between the course and the habit of skipping breakfast was found among boys: PR = 1.32 (95% CI = 1.09–1.59) in the fourth course of CSE (ISCED2); PR = 1.51 (95% CI = 1.20–1.90) in the second course of post-CSE (ISCED3); and PR = 1.81 (95% CI = 1.36–2.41) in the Intermediate Level Training Cycles (ISCED3), in comparison to the second course of CSE (ISCED2). Moreover, there was an association between skipping breakfast and the academic performance in both girls and boys. Among girls, there was a 78% (PR = 1.78; 95% CI = 1.48–2.13) and 135% (PR = 2.35; 95% CI = 1.90–2.91) increase in the risk of skipping breakfast for those with average and poor grades, respectively, in comparison to those with good grades. Similarly, in boys, the risk of not having breakfast was 51% (PR = 1.51; 95% CI = 1.14–2.00) and 100% (PR = 2.00; 95% CI = 1.49–2.68) higher for those with average grades and poor grades, respectively, compared to those with good grades. No statistically significant associations were found considering the migrant status, the size of the municipality or whether they were on a diet or not, for either girls or boys.

## 4. Discussion

The main findings of this study are that skipping of breakfast is associated with the female gender, lower socioeconomic position and a latter high-school course level. As for the latter parameter, the risk of skipping breakfast every day was 30% and 28% higher, respectively, among girls and boys from the most disadvantaged SEP, in comparison to those in the most advanced SEP. 

### 4.1. Prevalence of Skipping Breakfast and Socioeconomic Position (SEP) and Other Social Related Factors

The prevalence of skipping breakfast among adolescents in this study was higher than the 8% previously observed in the ANIBES study in Spain [33]. As reported by other studies, this prevalence was unequally distributed by SEP [17,34,35]. Socioeconomic inequalities in dietary behavior are persistent and widespread [36] and are contributing to inequalities in diet-related chronic diseases [37]. Indeed, in Spain, almost 50% of the general population is overweight; with individuals from disadvantaged SEP presenting the highest prevalence of obesity [38]. Moreover, nearly 30% of infants and children are already overweight [39]. Some studies have reported associations between skipping breakfast and overweight in children and adolescents [23,40,41]. These results confirm the importance of a continuous nutritional education and intervention in the obesogenic environments of the Spanish population, to promote awareness of healthy eating and facilitate an adequate breakfast among children and adolescents [33].

Several explanations for inequalities in dietary behavior have been proposed. First, individuals from a disadvantaged SEP, considering educational attainment, income levels, or occupation status, may lack the material and psychosocial resources that are typical of more advantaged SEPs. Indeed, material resources (higher food budgets and access to health-promoting goods and services [42]), and psychosocial resources (nutrition knowledge, cooking skills, and positive attitudes towards healthy eating [43,44]) are known to contribute to healthier dietary behavior, such as daily breakfast. In this line, eating behaviors are modelled by the family environment that includes not only food availability, but also parents’ eating behaviors [45]. Families from the most advantaged SEPs have a higher frequency of conversations on healthy food consumption in comparison to families from the most disadvantaged SEPs [46]. In the parents’ perspective, they lose authority over their children when they become adolescents and can no longer control their food preferences [47]. This happens especially among families from more disadvantaged SEPs, while middle SEP parents tend to direct the eating behavior of adolescents through a ’healthy direction’ [48]. The concern for a healthy diet ranks below other more pressing concerns for more disadvantaged SEP families [48]. Besides the family context, socio-cultural environments and peers influence teenagers’ lifestyles, play a role in food consumption. Some authors point out that is relevant to focus on modeling healthy eating behaviors among groups of teenage friends to promote healthy lifestyles among young people [49].

Likewise, SEP is a known determinant of the academic performance of adolescents [50]. The effects of inequalities of socioeconomic origin can be transmitted through very diverse mechanisms, such as different parental expectations, the availability of educational resources at home, or the influence of the socioeconomic position of peers [51,52]. Our results show an association between skipping breakfast and poor academic performance. In both sexes, there was an important increase in the risk of skipping breakfast for adolescents with medium and low grades, in comparison to those with good grades. Skipping breakfast due to sleeping late or lack of time may also reflect other overlapping risk factors that, in theory, contribute to a more chaotic or disorganized life, and this may also influence study and academic performance [53]. Addressing social inequalities and providing tools to organize and schedule time for breakfast could produce not only nutritional but also cognitive benefits [20,54].

Our findings also show that skipping breakfast has been associated with the female gender. Previously, gender differences have shown that girls skip breakfast more often than boys [34,54]. Although there are differences between the way we define breakfast, its variable, and the scale to measure the socioeconomic position, the conclusions reached are in line with the findings in the HBSC study as both studies find an association between breakfast consumption and socioeconomic position [17], the girls of lower SEP being those who skip breakfast the most. These data show us a largest gender gap in the most disadvantaged SEP. These differences in the frequency of breakfast consumption between boys and girls indicate that the influence of the family can be different depending on the gender. For instance, weight-control practices in girls have been observed to be partially modelled by mothers’ attitudes and behavior [55,56], and modeling seems to affect more girls than boys [57]. Therefore, considering a gender perspective in research and intervention programs can be fundamental for their design, content, messages, acceptability, and effectiveness [58].

Consistent with previous studies, we finally found that the prevalence of not eating breakfast seems to be greater among older adolescents [17,34]. This decrease could be related to an increased autonomy in food choices, and to a greater concern for body image, as observed in some studies [53,59]. However, in our study, we did not observe any relationship between skipping breakfast and dieting to lose or maintain weight, in line with other literature suggesting that the reasons for skipping breakfast were rarely weight-related [53]. Although more research would be needed to clarify the reasons for skipping breakfast, “lack of time” or “not being hungry early in the morning” are some of the reasons stated by adolescents [34,53,60]. This is also in line with the compaction of the morning schedule as the school level progresses. Eliminating afternoon classes and starting earlier in the morning may increase the feeling of lack of appetite and contribute to skipping breakfast. Also, the morning rush of adolescents and families may lead to increase the consumption of processed bakery products, resulting in a poor-quality breakfast. This happens especially if the skills for planning, preparing, and purchasing healthy options are low [61].

No relationship was found between the size of the municipality and the prevalence of breakfast skipping, in contrast to the study of Wadolowska et al. [34]. 

### 4.2. Strengths and Limitations

In our study, one potential limitation is that the data were self-reported, so there may be recall bias or inaccurate reporting of socioeconomic data. However, the use of self-reported questionnaires is a common method in this type of study, because of their low cost and easy administration [62]. A potential limitation could be how the dependent variable “skipping breakfast” was created. We classified participants into two categories never having breakfast versus having breakfast at least once a week, which is the most extreme situation. However, to analyze whether our results related to socioeconomic inequalities were driven by how the variable “skipping breakfast” was built, we conducted a sensitivity analysis. We repeated the multilevel regression analysis using as the dependent variable having breakfast three or fewer times a week versus four or more times (results shown on supplementary Table S1). The results were similar, but the associations were attenuated, which was expected since the dependent variable chosen in the study represents the most extreme situation in relation to skipping breakfast. Moreover, we have focused exclusively on having or not having breakfast every day, and not on possible differences between weekdays or weekends, nor on the composition or nutritional quality of this meal. Studies on these issues could be the subject of future research. Another limitation could be that in our study we measured perceived SEP instead of using a direct measure. However, the variable used comes from a validated scale with good psychometric properties. Moreover, several studies among adults and adolescents suggest that ladder rankings are more powerful determinants of health-related outcomes than traditional measures of SEP [63,64,65,66]. Despite these limitations, our study provides important information on the prevalence of skipping breakfast in a relatively large sample of secondary-school students from Central Catalonia (7319 students from 71% of the secondary education centers) including adolescents from rural areas. This allowed us to perform a strong statistical analysis and to find specific associations between variables and differentiate from studies with exclusively urban samples. Moreover, this study is the first wave of cohort, and could be the foundation for possible subsequent future interventions and a follow up in that population.

### 4.3. Implications and Recommendations

Strategies to promote healthy breakfast consumption among adolescents should be of diverse nature: policies, as well as, environmental, community, and family interventions. Moreover, those strategies should pursue sustainable long-term impacts [67].

The relationship of economic and social factors with breakfast consumption identified in the present paper and elsewhere should be tackled to ensure adolescents from different genders and backgrounds to benefit from specific actions [16]. 

At school level, actions should focus on primary school children but also on adolescents, since a high percentage of them tend to skip breakfast [16,54]. It is important to include nutrition education in the curriculum accompanied by the implementation of knowledge, skills, and attitudes for families that help to change habits and raise consciousness of the need for a daily healthy breakfast [67,68,69]. It is advisable to have breakfast at home before going to school and, if it is possible, shared with the family, and provide them tips for planning and preparing breakfast to overcome the barriers of possible lack of time. Breakfast interventions could be more effective by considering parental education level and also focusing on the peer social network [70]. Barriers and behaviors should be analyzed in the different school contexts [71,72].

Interventions on meal timing and frequency have been shown to be favorable since these aspects are the foundations for shaping eating behaviors [15]. As previously discussed, compact schedules probably do not facilitate the intake of breakfast before school. In some regions in Europe, political efforts are being made to push back the compacted school schedule that forces adolescents to rise early. Indeed, as previously specified, such s schedule could decrease sleeping hours and foster skipping breakfast or opt for ready to use unhealthy breakfast options [61]. Moreover, with compacted schedules, most of the canteens in schools disappear (profitability drops and they end up closing). Without a healthy food supply at school, the responsibilities of families and adolescents are very high and more complex. In other words, compacted schedules imply fewer meals at school. Meals at school contribute to assure a balanced daily dietary intake for adolescents from families with food insecurity and foster the role of the school to promote healthy habits. Young people with food insecurity are almost twice as likely to have fair or poor health [73]. In the food deserts in particular, where healthy foods are not easily accessible at an affordable price, it is common to have the unhealthiest breakfast options that include the most affordable food types [74]. Therefore, food-insecure households consume less fruit and vegetables and more unhealthy low-quality foods [75,76] (fast-food, pastries, etc.), in comparison with food-secure households. This may be related to weight gain and an increased risk of cardio metabolic complications in the future [23]. 

In addition to the initiatives discussed above at school and family level, actions should aim to reinforce effective programs that raise the likelihood of acquiring the breakfast habit along with the accessibility, availability, and consumption of healthy foods [50].

In conclusion, in the context of the current obesity epidemic, breakfast is the first meal of the day determining physical and intellectual functioning, and its regular intake is a relevant indicator of a healthy lifestyle [19]. Therefore, it is of high priority to keep on spreading and acting on the need for daily breakfast consumption, especially within secondary school environments of disadvantaged SEP. Rethinking schedules and controlling school food supply would be clear strategies for intervention in a facilitator healthy environment. Regular daily breakfast intake, fruit and vegetable consumption, and physical activity among young people are the most relevant aspects to prevent chronic diseases and the increase of the health gap across socioeconomic conditions and genders. 

## 5. Conclusions

The findings of the present investigation show socioeconomic and gender inequalities in breakfast consumption among adolescents in Spain. The risk of skipping breakfast was found to be 30% higher in girls and 28% higher in boys from the most disadvantaged SEP, in comparison to those from the more advanced SEP. Future public policies could be adapted considering a SEP and gender perspective to avoid increasing nutritional and health inequalities. 

## Figures and Tables

**Table 1:** Distribution of participants according to the independent variables. First wave of the DESKcohort project, 2020.

	Girls (*n* = 3814)		Boys (*n* = 3505)	
	*n*	%	*n*	%
Age [mean (SD)] *	15.3	1.7	15.3	1.7
**Course**				
2nd course of CSE	1382	36.2	1296	37.0
4th course of CSE	1374	36.0	1316	37.5
2nd course of PCSE	863	22.7	649	18.5
ILTC	195	5.1	244	7.0
SEP [mean (SD)] *	63.1	14.2	63.3	14.7
**Migratory status**				
Native	2850	74.7	2708	77.2
First- or second-generation immigrant	801	21.0	679	19.4
No data	163	4.3	118	3.4
**Size of municipality**				
≤5000r inhabitants	995	26.1	1015	29.0
5001r–20,000 inhabitants	1565	41.0	1350	38.5
>20,000 inhabitants	1147	30.1	1059	30.2
Living outside Central Catalonia	107	2.8	81	2.3
**Academic performance**				
Good grades	1128	29.6	884	25.2
Average grades	2208	57.9	2036	58.1
Poor grades	307	8.0	413	11.8
No data	171	4.5	172	4.9
**General health status**				
Excellent or very good	1911	50.1	2290	65.3
Good, fair or poor	1903	49.9	1215	34.7
Emotional state [mean (SD)] *	13.9	3.5	11.9	3.3
**Physical Activity**				
Over WHO recommendations	1303	34.2	1968	56.1
Under WHO recommendations	2200	57.7	1310	37.4
No data	311	8.1	227	6.5
**Body Mass Index**				
Underweight	92	2.4	120	3.4
Normal weight	2993	78.5	2503	71.4
Overweight or obesity	569	14.9	760	21.7
No data	160	4.2	122	3.5
**On a diet**				
No	3328	87.3	3132	89.3
Yes, to lose or maintain weight	187	4.9	136	3.9
Yes, for other reasons	299	7.8	237	6.8
**Having breakfast during last 7 days**				
Never	740	19.4	482	13.7
1 to 3 days	654	17.2	429	12.3
4 to 6 days	391	10.2	305	8.7
Every day	2029	53.2	2289	65.3

* In the variables age, socioeconomic position and emotional state, the heading “*n*” is substituted for “mean” and “%” for “SD”, Abbreviations: SD = standard deviation; CSE = compulsory secondary education (ISCED 2); PCSE = post-compulsory secondary education (ISCED3); ILTC = intermediate level training cycles (ISCED3); SEP = socioeconomic position.

**Table 2:** Prevalence of skipping breakfast every day in the last week for each of the independent variables.

	Girls			Boys		
	Skipping Breakfast Every Day			Skipping Breakfast Every Day		
	*n*	%	95% CI	*n*	%	95% CI
**Perceived SEP**						
Lowest SEP tertile	337	24.5	(22.3–26.9)	197	16.1	(14.2–18.3)
Medium SEP tertile	225	18.2	(16.1–20.4)	169	14.3	(12.4–16.4)
Highest SEP tertile	178	14.8	(12.9–17.0)	116	10.5	(8.8–12.5)
**Course**						
2nd course of CSE	205	14.8	(13.1–16.8)	137	10.6	(9.0–12.4)
4th course of CSE	275	20.0	(18.0–22.2)	188	14.3	(12.5–16.3)
2nd course of PCSE	204	23.6	(20.9–26.6)	106	16.3	(13.7–19.4)
ILTC	56	28.7	(22.8–35.5)	51	20.9	(16.3–26.5)
**Migratory status**						
Native	514	18.0	(16.7–19.5)	361	13.3	(12.1–14.7)
First- or second-generation immigrant	188	23.5	(20.7–26.5)	106	15.6	(13.1–18.5)
No data	38	23.3	(17.5–30.4)	15	12.7	(7.8–20.0)
**Size of municipality**						
≤5000r inhabitants	196	19.7	(17.3–22.3)	130	12.8	(10.9–15.0)
5001r–20,000 inhabitants	300	19.2	(17.3–21.2)	208	15.4	(13.6–17.4)
>20,000 inhabitants	221	19.3	(17.1–21.7)	126	11.9	(10.1–14.0)
Living outside Central Catalonia	23	21.5	(14.7–30.3)	18	22.2	(14.5–32.5)
**Academic performance**						
Good grades	123	10.9	(9.2–12.9)	79	8.9	(7.2–11.0)
Average grades	472	21.4	(19.7–23.1)	289	14.2	(12.7–15.8)
Poor grades	100	32.6	(27.6–38.0)	83	20.1	(16.5–24.2)
No data	45	26.3	(20.3–33.4)	31	18.0	(13.0–24.5)
**General health status**						
Excellent or very good	259	13.6	(12.1–15.2)	269	11.7	(10.5–13.1)
Good, fair or poor	481	25.3	(23.4–27.3)	213	17.5	(15.5–19.8)
**Emotional state**						
Highest tertile (better mood)	131	13.4	(11.4–15.7)	187	11.3	(9.8–12.9)
Medium tertile	219	17.8	(15.7–20.0)	157	14.1	(12.2–16.3)
Lowest tertile (worse mood)	390	24.3	(22.3–26.5)	138	18.9	(16.2–21.9)
**Physical Activity**						
Over WHO recommendations	211	16.2	(14.3–18.3)	226	11.5	(10.1–13.0)
Under WHO recommendations	458	20.8	(19.2–22.6)	216	16.5	(14.6–18.6)
No data	71	22.8	(18.5–27.8)	40	17.6	(13.2–23.1)
**Body Mass Index**						
Underweight	8	8.7	(4.4–16.4)	16	13.3	(8.3–20.7)
Normal weight	564	18.8	(17.5–20.3)	307	12.3	(11.0–13.6)
Overweight or obesity	136	23.9	(20.6–27.6)	136	17.9	(15.3–20.8)
No data	32	20.0	(14.5–26.9)	23	18.9	(12.9–26.8)
**On a diet**						
No	653	19.6	(18.3–21.0)	432	13.8	(12.6–15.0)
Yes, to lose or maintain weight	39	20.9	(15.6–27.3)	24	17.6	(12.1–25.0)
Yes, for other reasons	48	16.1	(12.3–20.7)	26	11.0	(7.6–15.6)
**Total**	**740**	**19.4**	**(18.2–20.7)**	**482**	**13.8**	**(12.7–14.9)**

Highlighted in bold the statistically significant associations. Abbreviations: CSE = compulsory secondary education (ISCED 2); PCSE = post-compulsory secondary education (ISCED3); ILTC = intermediate level training cycles; SEP = socioeconomic position.

**Table 3:** Prevalence ratios (PR) of skipping breakfast every day in the last week in 12- to 18-year-old adolescents from Central Catalonia, estimated using Poisson regression models.

	Girls				Boys			
	Crude PR		Adjusted PR		Crude PR		Adjusted PR	
	PR	95% CI	PR	95% CI	PR	95% CI	PR	95% CI
**Perceived socioeconomic position**								
Highest SEP tertile	**1.00**		**1.00**		**1.00**		**1.00**	
Medium SEP tertile	**1.22**	**(1.05–1.42)**	**1.10**	**(0.95–1.27)**	**1.36**	**(1.12–1.65)**	**1.25**	**(1.03–1.54)**
Lowest SEP tertile	**1.65**	**(1.42–1.92)**	**1.30**	**(1.12–1.52)**	**1.53**	**(1.25–1.87)**	**1.28**	**(1.04–1.58)**
**Course**								
2nd course of CSE	**1.00**		**1.00**		**1.00**		**1.00**	
4th course of CSE	**1.35**	**(1.13–1.62)**	**1.21**	**(1.00–1.45)**	**1.35**	**(1.11–1.64)**	**1.32**	**(1.09–1.59)**
2nd course of PCSE	**1.60**	**(1.34–1.91)**	**1.42**	**(1.19–1.71)**	**1.56**	**(1.25–1.96)**	**1.51**	**(1.20–1.90)**
ILTC	**1.94**	**(1.55–2.43)**	**1.52**	**(1.23–1.88)**	**1.97**	**(1.51–2.58)**	**1.81**	**(1.36–2.41)**
**Migratory status**								
Native	**1.00**				**1.00**			
First- or second-generation immigrant	**1.29**	**(1.13–1.49)**			**1.17**	**(0.92–1.48)**		
No data	**1.29**	**(1.01–1.65)**			**0.94**	**(0.54–1.63)**		
**Size of municipality**								
≤5000r inhabitants	**1.00**				**1.00**			
5001r–20,000 inhabitants	**0.96**	**(0.78–1.17)**			**1.20**	**(0.99–1.48)**		
>20,000 inhabitants	**0.98**	**(0.78–1.22)**			**0.93**	**(0.75–1.15)**		
Living outside Central Catalonia	**1.08**	**(0.71–1.63)**			**1.71**	**(1.13–2.58)**		
**Academic performance**								
Good grades	**1.00**		**1.00**		**1.00**		**1.00**	
Average grades	**1.96**	**(1.63–2.36)**	**1.78**	**(1.48–2.13)**	**1.59**	**(1.20–2.09)**	**1.51**	**(1.14–2.00)**
Poor grades	**2.98**	**(2.42–3.66)**	**2.35**	**(1.90–2.91)**	**2.25**	**(1.66–3.04)**	**2.00**	**(1.49–2.68)**
No data	**2.40**	**(1.65–3.49)**	**2.12**	**(1.47–3.06)**	**2.02**	**(1.35–3.04)**	**1.85**	**(1.22–2.79)**
**General health status**								
Excellent or very good	**1.00**		**1.00**		**1.00**			
Good, fair or poor	**1.86**	**(1.62–2.14)**	**1.48**	**(1.28–1.71)**	**1.49**	**(1.23–1.81)**		
**Emotional state**								
Highest tertile (better mood)	**1.00**		**1.00**		**1.00**		**1.00**	
Medium tertile	**1.33**	**(1.10–1.60)**	**1.17**	**(0.96–1.42)**	**1.25**	**(1.06–1.47)**	**1.14**	**(0.96–1.34)**
Lowest tertile (worse mood)	**1.81**	**(1.52–2.16)**	**1.33**	**(1.11–1.58)**	**1.67**	**(1.35–2.08)**	**1.35**	**(1.07–1.69)**
**Physical Activity**								
Over WHO recommendations	**1.00**				**1.00**		**1.00**	
Under WHO recommendations	**1.28**	**(1.06–1.53)**			**1.43**	**(1.23–1.66)**	**1.26**	**(1.07–1.48)**
No data	**1.41**	**(1.08–1.83)**			**1.53**	**(1.16–2.02)**	**1.40**	**(1.05–1.88)**
**Body Mass Index**								
Normal weight	**1.00**		**1.00**		**1.00**		**1.00**	
Underweight	**0.46**	**(0.24–0.86)**	**0.49**	**(0.26–0.91)**	**1.08**	**(0.62–1.91)**	**1.04**	**(0.60–1.81)**
Overweight or obesity	**1.26**	**(1.09–1.46)**	**1.09**	**(0.94–1.27)**	**1.45**	**(1.21–1.74)**	**1.42**	**(1.18–1.71)**
No data	**1.06**	**(0.76–1.48)**	**1.03**	**(0.75–1.43)**	**1.54**	**(0.98–2.42)**	**1.45**	**(0.93–2.24)**
**On a diet**								
No	**1.00**				**1.00**			
Yes, to lose or maintain weight	**1.06**	**(0.82–1.37)**			**1.27**	**(0.89–1.82)**		
Yes, for other reasons	**0.82**	**(0.61–1.10)**			**0.80**	**(0.55–1.15)**		

Highlighted in bold the statistically significant associations. Abbreviations: CSE = compulsory secondary education (ISCED 2); PCSE = post-compulsory secondary education (ISCED3); ILTC = intermediate level training cycles; SEP = socioeconomic position.

## Data Availability

The data presented in this study are available upon reasonable request to the corresponding author. The data are not publicly available due to confidentiality reasons.

## Supplementary Materials

The following are available online at https://www.mdpi.com/article/10.3390/nu13082500/s1, Table S1: Prevalence ratios (PR) of having breakfast 3 or less times in the last week in 12–18-years-old adolescents from Central Catalonia, estimated using Poisson regression models.
