# Supplementary material for: Social Inequalities in Breakfast Consumption among Adolescents in Spain: The DESKcohort Project
Source: Nutrients. 2021 Jul 22;13(8):2500. doi: 10.3390/nu13082500 (PMC8401108; doi:10.3390/nu13082500)
Supplement: Supplementary file 1 [file nutrients-13-02500-s001.zip › nutrients-1263367-supplementary.pdf]

Supplementary Material

Table S1. Prevalence ratios (PR) of having breakfast 3 or less times in the last week in 12-18-years-old adolescents from Central Catalonia, estimated using Poisson regression models.

	Girls				Boys			
	Crude PR		Adjusted PR		Crude PR		Adjusted PR	
	PR	95%CI	PR	95%CI	PR	95%CI	PR	95%CI
**Perceived socioeconomic position**								
Highest SEP tertile	1.00		1.00		1.00		1.00	
Medium SEP tertile	**1.14**	**(1.02-1.29)**	1.05	(0.93-1.17)	**1.24**	**(1.24-1.41)**	**1.13**	**(1.00-1.28)**
Lowest SEP tertile	**1.44**	**(1.29-1.60)**	**1.17**	**(1.06-1.30)**	**1.37**	**(1.37-1.55)**	1.12	(0.99-1.27)
**Course**								
2nd course of CSE	1.00		1.00		1.00		1.00	
4th course of CSE	**1.22**	**(1.09-1.37)**	1.11	(0.99-1.24)	**1.28**	**(1.28-1.46)**	**1.25**	**(1.09-1.43)**
2nd course of PCSE	**1.27**	**(1.14-1.42)**	**1.15**	**(1.02-1.28)**	**1.38**	**(1.38-1.63)**	**1.36**	**(1.13-1.64)**
ILTC	**1.61**	**(1.41-1.83)**	**1.32**	**(1.14-1.51)**	**1.89**	**(1.89-2.23)**	**1.72**	**(1.45-2.03)**
**Migratory status**								
Native	1.00		1.00		1.00		1.00	
First- or second-generation immigrant	**1.37**	**(1.26-1.49)**	**1.24**	**(1.15-1.33)**	**1.36**	**(1.36-1.55)**	**1.22**	**(1.08-1.38)**
No data	**1.26**	**(1.09-1.47)**	1.12	(0.96-1.31)	**1.08**	**(1.08-1.48)**	0.97	(0.72-1.32)
**Size of municipality**								
≤5,000 inhabitants	1.00				1.00			
5,001-20,000 inhabitants	1.07	(0.95-1.20)			**1.16**	**(1.16-1.33)**		
>20,000 inhabitants	1.06	(0.93-1.22)			**1.01**	**(1.01-1.18)**		
Living outside Central Catalonia	1.01	(0.76-1.33)			**1.79**	**(1.79-2.44)**		
**Academic performance**								
Good grades	1.00		1.00		1.00		1.00	
Average grades	**1.56**	**(1.38-1.77)**	**1.40**	**(1.24-1.59)**	**1.53**	**(1.53-1.88)**	**1.44**	**(1.17-1.76)**
Poor grades	**2.16**	**(1.87-2.49)**	**1.71**	**(1.48-1.99)**	**1.99**	**(1.99-2.48)**	**1.74**	**(1.42-2.14)**
No data	**1.94**	**(1.61-2.33)**	**1.64**	**(1.36-1.97)**	**1.80**	**(1.80-2.37)**	**1.58**	**(1.21-2.06)**
**General health status**								
Excellent or very good	1.00		1.00		1.00		1.00	
Good, fair or poor	**1.65**	**(1.53-1.78)**	**1.38**	**(1.27-1.51)**	**1.48**	**(1.48-1.67)**	**1.22**	**(1.07-1.39)**
**Emotional state**								
Highest tertile (better mood)	1.00		1.00		1.00		1.00	
Medium tertile	**1.28**	**(1.12-1.47)**	**1.19**	**(1.04-1.37)**	**1.20**	**(1.20-1.33)**	1.08	(0.96-1.21)
Lowest tertile (worse mood)	**1.69**	**(1.49-1.91)**	**1.39**	**(1.22-1.57)**	**1.52**	**(1.52-1.78)**	**1.23**	**(1.03-1.47)**
**Physical Activity**								
Over WHO recommendations	1.00				1.00		1.00	
Under WHO recommendations	**1.26**	**(1.13-1.41)**			**1.40**	**(1.40-1.56)**	**1.20**	**(1.07-1.36)**
No data	**1.44**	**(1.24-1.68)**			**1.39**	**(1.39-1.68)**	**1.26**	**(1.03-1.54)**
**Body Mass Index**								
Normal weight	1.00				1.00		1.00	
Underweight	0.83	(0.58-1.19)			0.79	(0.79-1.14)	0.76	(0.53-1.10)
Overweight or obesity	**1.27**	**(1.16-1.40)**			**1.28**	**(1.28-1.46)**	**1.18**	**(1.03-1.35)**
No data	1.01	(0.81-1.26)			**1.45**	**(1.45-1.88)**	1.30	(1.00-1.68)
**On a diet**								
No	1.00				1.00			
Yes, to lose or maintain weight	1.14	(0.98-1.33)			**1.33**	**(1.33-1.63)**		
Yes, for other reasons	0.91	(0.74-1.13)			0.79	(0.79-1.08)		

Highlighted in bold the statistically significant associations. Abbreviatures: CSE=compulsory secondary education (ISCED 2); PCSE=post-compulsory secondary education (ISCED3); ILTC=intermediate level training cycles; SEP=socioeconomic position.
